# Supplementary material for: Regular Exercise in Drosophila Prevents Age-Related Cardiac Dysfunction Caused by High Fat and Heart-Specific Knockdown of skd
Source: Int J Mol Sci. 2023 Jan 7;24(2):1216. doi: 10.3390/ijms24021216 (PMC9865808; doi:10.3390/ijms24021216)
Supplement: Supplementary file 1 [file ijms-24-01216-s001.zip › ijms-2077783-supplementary.pdf]

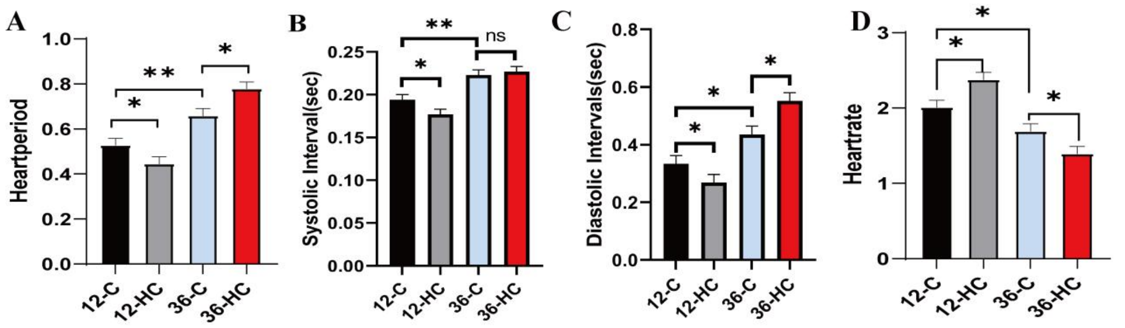

Figure S1. Effects of HFD and aging on cardiac function. (A) Cardiac function assays included HP in 12-C, 12-HC, 36-C and 36-HC group. (B) Cardiac function assays included SI in 12-C, 12-HC, 36-C and 36-HC group. (C) Cardiac function assays included DI in 12-C, 12-HC, 36-C and 36-HC group. (D) Cardiac function assays included HR in 12-C, 12-HC, 36-C and 36-HC group, N = 25 ± 5. ,Note: N = 25 ± 5, ns P > 0.05; * P < 0.05; ** P < 0.01.

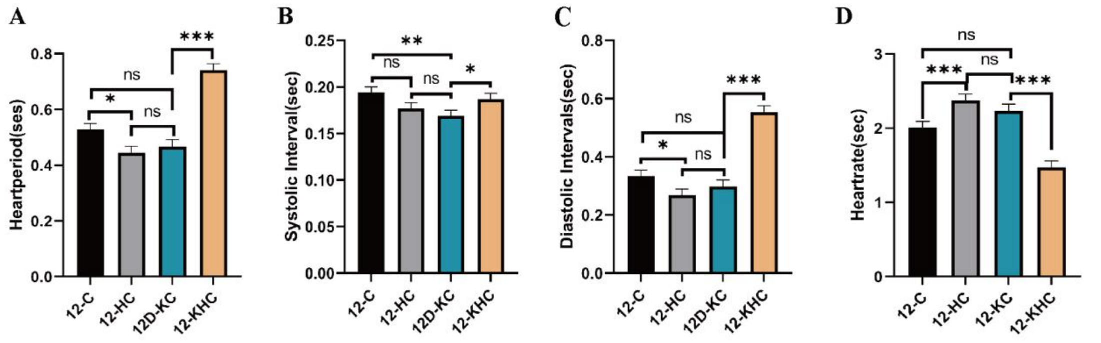

Figure S2. Cardiac function in 12-KC *Drosophila* under NFD and HFD conditions. (A-D) Effects of regular exercise on HP, SI, DI and HR in cardiac function in 12D Hand-Gal$_4$>*skd* RNAi NFD and HFD *Drosophila*. Note: N = 25 ± 5, ns P > 0.05; * P < 0.05; ** P < 0.01; ***P < 0.001.

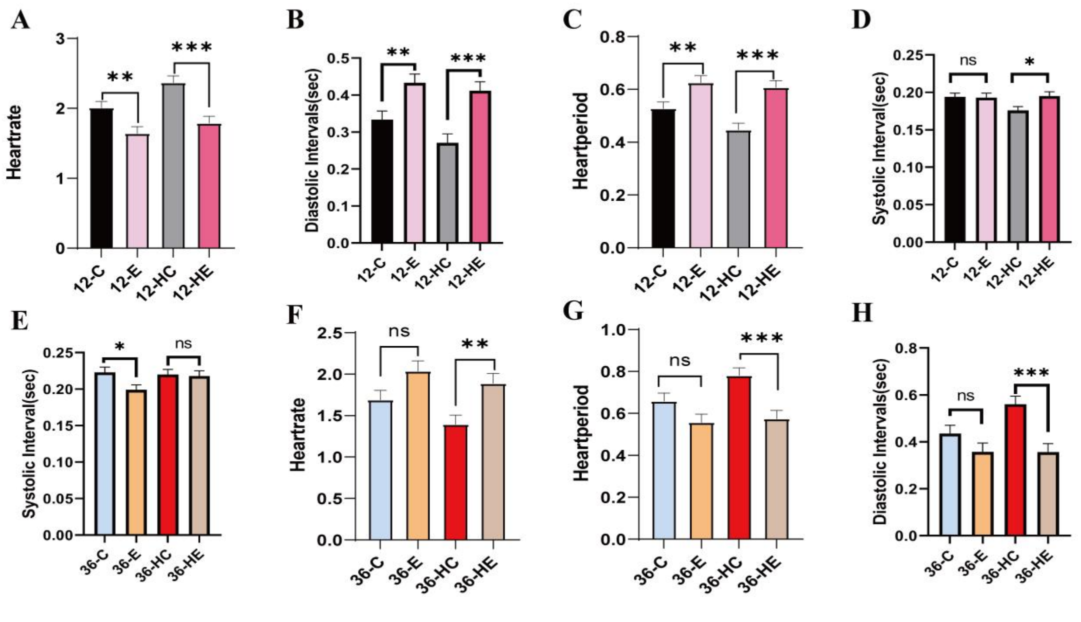

Figure S3. Effects of regular exercise on cardiac function of *Drosophila* with HFD and aging. (A-D) Regular exercise affects HR, DI, HP and SI in cardiac function in 12D NFD and HFD *Drosophila*. (E-H) Regular exercise affects SI, HR, HP and DI in cardiac function in 36D NFD and HFD *Drosophila*. Note: N = 25 ± 5, ns P > 0.05; * P < 0.05; ** P < 0.01; ***P < 0.001.

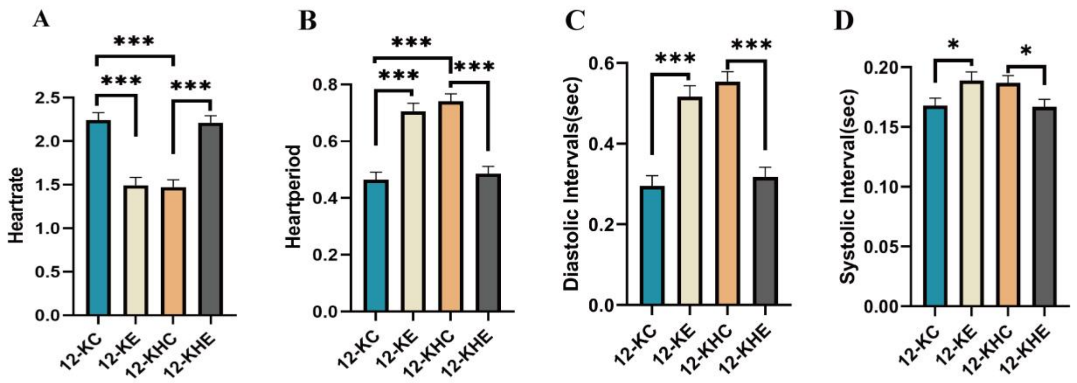

Figure S4. Effects of regular exercise on cardiac function in Hand-Gal$_4$>*skd* RNAi HFD *Drosophila*. (A-D) Effects of regular exercise on HR, HP, DI and SI in cardiac function in 12D Hand-Gal$_4$>*skd* RNAi NFD and HFD *Drosophila*. Note: N = 25 ± 5, ns P > 0.05; * P < 0.05; ***P < 0.001.
